# Jump-starting life: balancing transposable element co-option and genome integrity in the developing mammalian embryo

**DOI:** 10.1038/s44319-024-00118-5

**Published:** 2024-03-25

**Authors:** Marlies E Oomen, Maria-Elena Torres-Padilla

**Affiliations:** 1 Institute of Epigenetics and Stem Cells, Helmholtz Zentrum München, München, Germany; 2 Faculty of Biology, Ludwig-Maximilians Universität, München, Germany

**Keywords:** Transposable Elements, Genome Evolution, Preimplantation Development, Genome Regulation, Chromatin, Transcription & Genomics, Development, Stem Cells & Regenerative Medicine

## Abstract

Remnants of transposable elements (TEs) are widely expressed throughout mammalian embryo development. Originally infesting our genomes as selfish elements and acting as a source of genome instability, several of these elements have been co-opted as part of a complex system of genome regulation. Many TEs have lost transposition ability and their transcriptional potential has been tampered as a result of interactions with the host throughout evolutionary time. It has been proposed that TEs have been ultimately repurposed to function as gene regulatory hubs scattered throughout our genomes. In the early embryo in particular, TEs find a perfect environment of naïve chromatin to escape transcriptional repression by the host. As a consequence, it is thought that hosts found ways to co-opt TE sequences to regulate large-scale changes in chromatin and transcription state of their genomes. In this review, we discuss several examples of TEs expressed during embryo development, their potential for co-option in genome regulation and the evolutionary pressures on TEs and on our genomes.

## Abstract (web summary)

This review discusses examples of transposable element (TE) activation and mechanisms of TE co-option for genome regulation in the early embryo, germline, placenta and embryonic stem cells.

## Graphical abstract

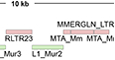

## Introduction

Initially discovered in the 1940s by Barbara McClintock in maize, we now know that transposable elements (TEs) are present in large numbers throughout the great diversity of eukaryotic genomes (McClintock, 1950; Osmanski et al, 2023). At the start of the genomics era, these highly repetitive elements were often regarded as “junk DNA”, genomic elements with no apparent function (Lander et al, 2001). While TEs are, by definition, selfish elements that colonize our genomes, there are now several examples which demonstrate that our genomes were able to domesticate TE sequences for the benefit of the host species, a process also known as co-option (Sundaram and Wysocka, 2020; Fueyo et al, 2022).

Expression of many TE insertions and families is a hallmark of early embryonic development but has also been associated with specific cell types at later stages of mammalian development, such as both the male and female germline and the placenta, as well as cultured embryonic stem cell lines (Zamudio and Bourc’his, 2010; Hackett et al, 2017; Chuong, 2013; Peaston et al, 2004). Perhaps unsurprisingly, these tissues and cells are known to have more naïve (open) chromatin than somatic differentiated cell types, and are typically characterized by bivalent histone marks at developmental genes and incomplete establishment of cannonical heterochromatin marks (Gaspar-Maia et al, 2011; Meshorer and Plath, 2020; Hemberger and Dean, 2023; Fu et al, 2020; Saitou and Yamaji, 2012; Burton and Torres-Padilla, 2014; Vastenhouw and Schier, 2012). The fact that TEs become also highly expressed in some cancer cells has contributed to our perception that their expression is harmful for the organism, adding to the increased genome instability and gene misregulation (Burns, 2017). In a developmental context, TE expression is not simply a consequence of the genome-wide reorganization of chromatin. Their expression is widespread across all insertions and TE families, and the expression of a handful of individual TE families has been demonstrated to be essential for proper progression of embryonic development in various mammalian species (Sakashita et al, 2023; Modzelewski et al, 2021; Jachowicz et al, 2017). Despite observations of both beneficial as well as harmful consequences of TE expression, it must be noted that the majority of TE insertions in our genome are neutral, either because they are silenced by the host genome or neutralized by decay of sequence integrity over evolutionary time.

The exponential growth of available high-throughput sequencing data and the ongoing efforts to assemble and annotate the genomes of a large number of species, allow us to learn more about these two-faced elements (Storer et al, 2021; Osmanski et al, 2023). Both the transcriptional regulation of TEs by their host genomes as well as how TEs themselves influence gene regulation of the host genomes are the research focus of many scientists. In this review we aim to highlight the variety of TEs, showcase several examples of TEs that are expressed during mammalian development and discuss the different evolutionary pressures on TEs as well as their co-option by the host genome.

## TEs and their remnants in our genomes

**Figure 1:**
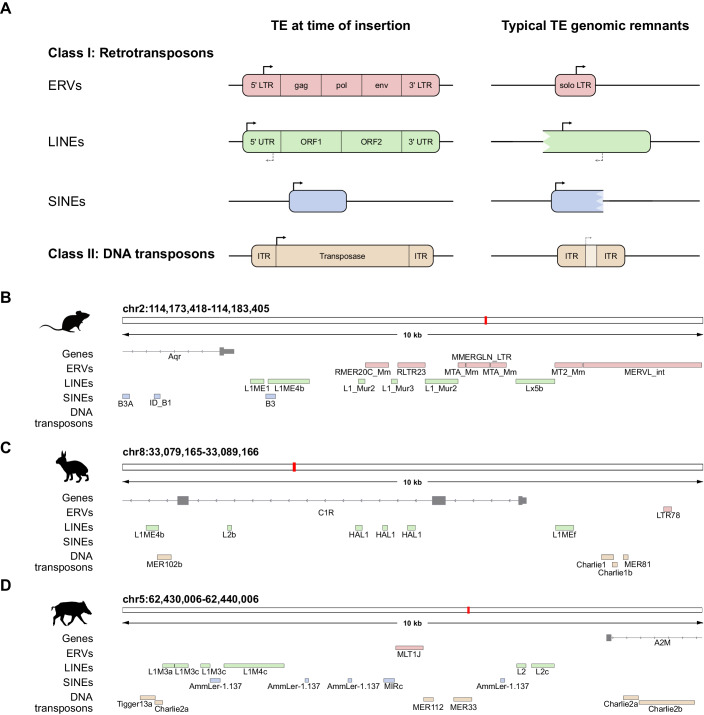
TE characteristics in host genomes at time of insertion and their remnants. (**A**) TE sequences can be classified as retrotransposons (Class I; ERVs, LINEs, and SINEs) and DNA transposons (Class II) based on their transposition mechanism and sequence features. Although at time of insertion the different TE subclasses had very distinct characteristics, the sequences that can be found more commonly in most mammalian genomes are much shorter, with only certain identifying features remaining. (**B**–**D**) TE sequences and their remnants are present in high copy numbers throughout mammalian genomes, as shown here in example regions of the mouse (**B**), rabbit (**C**), and pig genomes (**D**).

TEs are typically classified in two major classes: class I, which includes retrotransposons, and class II, which includes DNA transposons (Finnegan, 1992, 1989; Wells and Feschotte, 2020; Storer et al, 2021), with retrotransposons representing the vast majority of TE insertions in mammalian genomes (Rodriguez-Terrones and Torres-Padilla, 2018; Osmanski et al, 2023; Lander et al, 2001). While most DNA transposons excise themselves in order to reintegrate at another position in the genome (cut and paste), retrotransposons use an RNA intermediate for transposition events (copy and paste), which allowed them to quickly multiply throughout the genome (Finnegan, 1989). Retrotransposons can be further grouped into 3 main subclasses; Long-Terminal Repeat (LTR) containing elements (which comprise mainly, but not exclusively, ERVs (endogenous retroviral elements)), LINEs (long interspersed nuclear elements) and SINEs (short interspersed nuclear elements), based on their origin, their transposition strategy and sequence structures (Fig. 1A) (Wells and Feschotte, 2020). Beyond the subclasses, TEs can be further classified in superfamilies, with examples such as the group of LINE elements LINE1, LTRs ERVL-MaLR and SINE elements Alu. Lastly, TEs are annotated as families, comprised of sets of TE insertions (individual genomic locations) with high sequence similarity that are assumed to originate from the same ancestral transposing element, such as the primate-specific ERVL LTR element MLT2A1 and mouse-specific MT2_Mm, which are discussed in greater detail below. Recent studies have further characterized families of TEs in subfamilies based on phylogenetic analyses of their sequence divergence, of which the human ERV family LTR7 is an example (Carter et al, 2022).

Following the structural characterization of subclasses, full-length ERVs typically consist of 3 open reading frames (ORFs): gag, pol, env, which encode the viral proteins required for transposition and viral particle formation, flanked by LTRs on both sides, which drive their transcription (Wells and Feschotte, 2020) (Fig. 1A). Most ERVs have lost the envelope protein env, which is essential for reinfection and integration of new cells, while still maintaining the ability to retrotranspose within the same cell (Magiorkinis et al, 2012). Full-length LINEs contain two ORFs and possess either a monomer-based regulatory 5’ region, for example in the case of mice, or a 5’UTR region, for example in primates. ERVs and LINEs are classified as autonomous retrotransposons as their full-length sequences posses all information essential for transposition (Wells and Feschotte, 2020; Jachowicz and Torres-Padilla, 2016; Belancio et al, 2009). SINEs, however, rely on the transposon machinery of other retrotransposons, most often LINEs, and are therefore referred to as non-autonomous retrotransposons (Wells and Feschotte, 2020; Storer et al, 2021; Smit et al, 1995; Kramerov and Vassetzky, 2011; Khan et al, 2006; Dewannieux et al, 2003). As a result, SINEs are a much more diverse subclass of retrotransposons compared with LINEs and LTRs, and contain several unique sequence structural and regulatory elements. Interestingly, the diverse origins of SINEs are also reflected in their transcription: a subgroup of SINEs can be transcribed by RNA polymerase III, reflecting the tRNA origin of certain SINEs, while all other retrotransposons instead use RNA polymerase II, which transcribes mostly mRNAs (Carnevali and Dieci, 2017).

As mentioned above, transposition of DNA transposons does not require an RNA intermediate. However, it does require the transcription and translation of a functional transposase from its coding region (Pace and Feschotte, 2007; Tan et al, 2021). The transposase allows the DNA transposon sequence to excise from the original location in the genome and reinsert itself in a second location. The coding region of the transposase gene is generally flanked by two regulatory inverted terminal repeats (ITRs) (Fig. 1A). Despite most commonly following a cut-and-paste strategy, DNA transposons have successfully invaded and multiplied throughout several mammalian genomes. One interesting model explaining such an efficient increase in their genomic copy number is based on the concordance of transposition events with S-phase, which could lead to the relocation of a DNA transposon from a replicated region into an non-replicated region, effectively resulting in a duplication upon completion of DNA replication (Wells and Feschotte, 2020; Ros and Kunze, 2001; Muñoz-López and García-Pérez, 2010; Tan et al, 2021).

Although the original sequence features and mode of transposition of TEs is an interesting field of study, the majority of TEs in mammalian genomes are no longer able to transpose, as they have lost many of the sequence features which allowed them to do so (Fueyo et al, 2022; Wells and Feschotte, 2020). What remains in our genomes are the remnants of their sequences (Fig. 1A). It is important to note that although many TEs have lost their ability to transpose, they have not lost their ability to be transcribed (see Box 1). This leaves traces of regulatory sequences with potential transcriptional activity scattered throughout the genome. For example, ERVs seem to have often lost their internal protein coding sequences (gag, pol, and env), leaving either both 5’ and 3’ LTRs or simply an individual solo LTR as most prevalent remnants in the genome (Fig. 1A). Intriguingly, these TE remnants are not rare or unique loci, but instead are very frequent occurrences throughout mammalian genomes (Rodriguez-Terrones and Torres-Padilla, 2018). As exemplified in Fig. 1B–D, TEs of all classes are located proximal and distal to genes but also within gene introns (Fueyo et al, 2022). In addition, although all TE classes and subclasses are present throughout the mammalian kingdom, the frequency and ratio of the TE insertions present in the genomes can vary widely (Rodriguez-Terrones and Torres-Padilla, 2018; Osmanski et al, 2023). Similarly, while many TE families and their remnants harbored in mammalian genomes are specific to a certain species or genus, there are ancient TEs that are shared across orders and even mammalian clades, and are therefore of much older evolutionary age (Storer et al, 2021; Osmanski et al, 2023; Matsushima et al, 2024).

Box 1 Transposition potential versus transcription potential The *transposition* potential of a TE and their *transcription* potential refer to two fundamentally different TE features. While many TEs have maintained transcription potential, only few TEs are known to have maintained their ability to (retro-)transpose, most notably LINE1 elements and Alu elements, a SINE subgroup (Dewannieux et al, 2003; Kazazian et al, 1988; Richardson et al, 2017). The maintained, or in some instances regained, transposition potential often leads to reduced genome integrity or loss and misregulation of genes, and is therefore typically associated with disease. Whether the actual disease state is caused by individual novel TE insertions inside or in close proximity of a gene, or is the result of a general genome-wide response to reduced genome stability, remains to be seen. In this review, we focus on the co-option of transcriptionally active TEs and refer the reader to other reviews on the topic of transposition activity of TEs in disease (Levin and Moran, 2011; Belancio et al, 2009; Kazazian and Moran, 2017; Burns, 2017; O’Donnell and Burns, 2010; Solyom and Kazazian, 2012).

## Transcriptional activation of TEs during development

**Figure 2:**
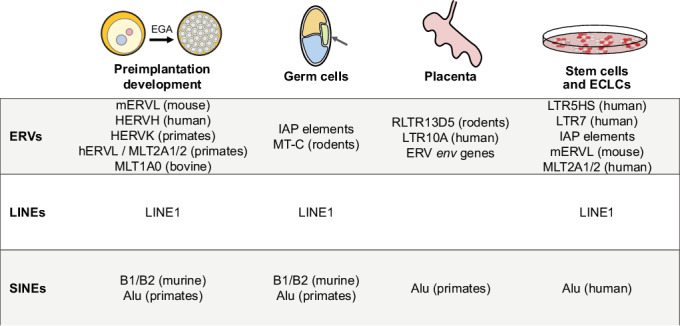
Examples of retrotransposon expression throughout developmental time. Examples of TEs from the different retrotransposon subclasses (ERVs, LINEs, and SINEs) expressed in the preimplantation embryo, germ cells, placenta, and in ESCs of mammalian systems.

During preimplantation development, the embryo activates its own genome after a period of transcriptional silencing in the male and female germline. Together with the degradation of maternally inherited transcripts, embryonic genome activation (EGA) is the main molecular process constituting the maternal-to-zygotic transition (MZT). In mammals, this coincides with a dramatic remodeling of chromatin. Furthermore, this key event in early mammalian development occurs prior to the establishment of mature heterochromatin (Burton et al, 2020), resulting in a transcriptionally permissive environment. TEs representing all subclasses become expressed at this developmental time. While it was initially thought that the wave of expression of TEs was an opportunistic, non-specific event linked to global heterochromatin remodeling in the early embryo, the notion that expression of TEs is simply a result of this naïve, permissive chromatin state has started to change. Instead, the patterns of TE expression are class and stage-specific, indicating a precise regulation. Moreover, not all TE families become expressed equally, but instead a specific subset of TEs are known to become transcribed. In particular, the transcriptional activity of ERVL, MaLR, and LINE1 elements are a key characteristic of mammalian preimplantation development (Fig. 2) (Hendrickson et al, 2017; Halstead et al, 2020; Peaston et al, 2004; Svoboda et al, 2004), and in some cases have been found essential for the progression of embryonic development (Sakashita et al, 2023; Jachowicz et al, 2017).

### Transcriptional activation of retrotransposons in the mammalian preimplantation embryo

The mammalian preimplantation embryo presents ideal conditions for TEs to become reactivated. Although for some TE families, it has been observed that only a small proportion of all the insertions in the genome are expressed (Modzelewski et al, 2021), for many others it has been suggested that many insertions become expressed simultaneously (Peaston et al, 2004). Most notably, a large fraction of elements from the mouse-specific mERVL and the human equivalent hERVL families are expressed at similar EGA timing in the two species and are regulated by the transcription factor DUX and its orthologue DUX4, respectively (Sakashita et al, 2023; Hendrickson et al, 2017; De Iaco et al, 2017; Peaston et al, 2004). Similar associations of TE expression during EGA mediated by specific transcription factors have been found for the transcription factors OBOX and Stella (Ji et al, 2023; Huang et al, 2017). This suggests a degree of conservation between the expression of these ERVL elements and their regulatory proteins across mammalian species. It is important to stress that hERVL and mERVL are species-specific elements, which do not belong to the same TE family, suggesting that the conserved transcriptional regulation of these elements is the result of convergent evolution. Several additional ERVs such as hERVH, hERVK and the hERVL LTRs MLT2A1 and MLT1A2 in human and other primates (Hashimoto et al, 2021; Carter et al, 2022), as well as ERVL elements from the MaLR family ORR1A0 in mouse (Franke et al, 2017) and MLT1A0 in bovine embryos (Halstead et al, 2020) are highly expressed at and around the time of EGA in early embryos. However, the precise TFs that modulate the expression patterns of these TEs are still largely unkown (Hermant and Torres-Padilla, 2021).

Although most studies have focused on activation of ERVs during EGA, several LINEs and SINEs are also expressed in the preimplantation embryo. For example, LINE1 elements, particularly those belonging to the Gf, Tf and A families, are expressed in the early mouse embryo. Interestingly, manipulating expression of such LINE1s results in changes of global genome accessibility, which we will describe in more detail below (Fadloun et al, 2013; Jachowicz et al, 2017). Although SINE elements tend to belong to evolutionary younger TE families and therefore show a more species-specific behavior, their expression can be found across mammalian species. Among others, SINEs B1 and B2 are expressed in mouse preimplantation embryos (Peaston et al, 2004; Fadloun et al, 2013), as well as Alu elements in early primate embryos (Jordà et al, 2017). Taken together, although TE families that have been reported to be expressed in early embryos are often specific for a given species or clade, activation of all main retrotransposons can be found in all mammalian species investigated. This suggests that the transcriptional activation of transposable elements is a conserved developmental hallmark of mammalian preimplantation development.

### Retrotransposon activation in the germline

In addition to the preimplantation embryo, TEs are also expressed in germ cells and their precursors, the primordial germ cells (Peaston et al, 2004; Gagnier et al, 2019; Ishiuchi and Torres-Padilla, 2014; Garcia-Perez et al, 2016; Zamudio and Bourc’his, 2010). During mammalian meiosis, certain TEs are transcriptionally active (van der Heijden and Bortvin, 2009). Interestingly, de novo germline mutations in the mouse have been traced back to transcription and transposition activity of specific TEs leading to novel TE insertions (Gagnier et al, 2019). The germline has evolved a unique defense mechanism to repress the expression of TE elements that is largely based on RNA entities, including small interfering RNAs (siRNA) and PIWI-associated RNAs (piRNA) (see Box 2) (Wang and Lin, 2021; Ozata et al, 2019). Nonetheless, some TEs escape these silencing mechanisms. These include TEs exclusively expressed in the germline, such as LTR MT2-C and IAP elements in rodents, as well as some TE superfamilies broadly expressed in the early developing embryo, such as LINE1, murine SINEs B1 and B2 and the primate SINE Alu (Zamudio and Bourc’his, 2010). The germline in particular is highly sensitive to the consequences of TE activity, and thus an intriguing tissue to study how cells balance both the beneficial and detrimental effects of transciptional and transpositional activation of TEs, as these will be passed on directly to the next generation.

Box 2 Silencing TEs and their transcripts The timely silencing of TEs is also an essential process in embryo development (Trono, 2017; Yang et al, 2017; Burton et al, 2020). One way this is established and maintained is through DNA methylation and the acquisition of heterochromatic histone methylation marks such as H3K9me3, typically guided by KRAB zinc finger proteins (Burton and Torres-Padilla, 2014; Wang et al, 2018; Almeida et al, 2022; Trono, 2017; Friedli and Trono, 2015; Rowe and Trono, 2011). Noteworthy, the germline has a unique alternative defense mechanism to modulate the levels of TE transcripts using PIWI-interacting RNAs (piRNAs). piRNAs have been observed both in mammalian systems as well as other non-vertebrate model organisms such as *Drosophila* and C*. elegans*, suggesting strong conservation of the PIWI pathway for TE regulation (Huang et al, 2013; Brennecke et al, 2007; Das et al, 2008; Chang et al, 2019; Girard et al, 2006; Aravin et al, 2006). The presence of piRNAs is typically followed by the accumulation of repressive chromatin modifications such as H3K9me3 and DNA methylation at those piRNA producing loci, which in turn leads to transcriptional repression of TEs (Le Thomas et al, 2013; Aravin et al, 2008). Moreover, it has been shown across model systems that mutants in the piRNA pathway are either fully sterile or subfertile. While the phenotype varies depending on the species and whether it is the male or the female germline, overall these findings suggest that proper silencing of TEs is essential for fertility (Wu et al, 2020; Aravin et al, 2006; Girard et al, 2006; Lau et al, 2006; Grivna et al, 2006).

### Retrotransposon activation in the placenta

In mammals, the first cell fate decision occurs during preimplantation development, leading to the segregation of the first two embryonic lineages. The inner cell mass gives rise to all of the embryonic lineages and to extra-embryonic components such as the yolk sac. The second lineage, the trophectoderm, comprises the precursor cells of the embryonic placenta. Thus, the cells of the trophectoderm will not contribute to the germline and therefore potential genetic changes occurring in the trophectoderm or the placenta will not be inherited by the next generation. This makes the placenta an atypical tissue for the activation of selfish elements, such as TEs, as new transposition events cannot be passed onto the progeny. However, several TEs, mostly LTRs/ERVs, are transcriptionally active in the placenta and its precursor trophoblast cells (Chuong, 2018). It has been suggested that the co-option of both TE transcripts as well as the proteins originating from TE encoded genes enabled the rapid evolution of the placenta as well as the large diversity in the mechanisms underlying the development of the placenta across eutherian mammals (see also Box 3) (Chuong, 2013). Specifically, LTR insertions of ERV element RLTR13D5 function as enhancer sequences of genes expressed in the placenta during mouse placental development in a species-specific manner (Chuong et al, 2013). Along these lines, in human trophoblast cells, the primate ERVL LTR element LTR10A and several other ERV elements are marked by H3K27ac, a histone modification typically found at enhancers (Frost et al, 2023). In addition, genes closely located to LTR10A sequences are typically upregulated when the neighboring TE is transcriptionally active, hinting towards gene regulation modulated by these TEs or, alternatively, to parallel activation of both the TE and the neighboring host gene (Frost et al, 2023). Lastly, the placenta-specific gene *syncytin* originates from the ERV *env* coding gene of the human ERV element HERV-W (Mi et al, 2000) (see also Box 3), showing that not only do ERVs become expressed, but can also be translated. In this particular example, SYNCYTIN7-1 has a role during placental morphogenesis, where it is essential for establishing the interface between the embryo and uterus (Mi et al, 2000). Indeed, reduced SYNCYTIN-1 expression has been correlated with preeclampsia in humans (Ruebner et al, 2013).

In contrast to these reports of reactivation of ERV elements, not much is known about the transcriptional activation of LINEs and SINEs in placental cells. However, SINE retrotransposition events have been implicated in the rapid multiplication and diversification of neighboring genes (Jurka et al, 2005). One key example is the duplication of placenta growth hormones, which were retrotransposed along with SINE elements of the Alu superfamily in primates (Barsh et al, 1983; Haig, 2008; Emera and Wagner, 2012). Taken together, placental development shows several examples of (often species-specific) co-option of TE sequences and translated TE proteins, reaffirming that TE sequences can be a resource to the host, allowing for rapid evolutionary innovation.

Box 3 TE proteins and their co-option In some cases, TE transcripts are translated into functional proteins. Generally, this leads to the ability to (retro-)transpose the activated TE family, as well as potential disease phenotypes (Kazazian and Moran, 2017; Levin and Moran, 2011; Belancio et al, 2009; Wood and Helfand, 2013; Burns, 2017). For many proteins encoded by TE transcripts, their role and how they continue to escape transcriptional and translational silencing remain unclear. Recently, it was shown that certain reactivated and translated ERVs maintain the ability to form viral particles in mouse early embryos and in stem cells derived from bats (Déjosez et al, 2023; Ribet et al, 2008). In rare cases, however, the proteins translated from TE sequences have been shown to be adapted by the host and to contribute to the function of a healthy cell. Notably, a recent publication has shown that the ERV-derived retroviral protein *SUPYN* is expressed in the human preimplantation embryo and developing placenta, where it has anti-viral properties (Frank et al, 2022). Similarly, the *env* coding gene of ERV elements (ERV *env*) contributes to the *syncytin* genes, which are essential genes exclusively expressed in placental and their precursor trophoblast cells and are conserved throughout eutherian and marsupial species (Chuong, 2013, 2018; Cornelis et al, 2015; Keighley et al, 2023; Emera and Wagner, 2012; Mi et al, 2000). Interestingly, this TE co-option for placental development is highly species-specific, resulting from convergent evolution (Chuong, 2013). This further emphasizes the rich variety of regulatory elements that TE sequences contribute to genome evolution.

### Transcriptional activation of retrotransposons in embryonic stem cells and 2-cell-like cells (2CLCs)

Embryonic stem cell (ESCs) lines derived from the inner cell mass of the mouse blastocyst have been widely used to study TE expression. Similarly, the relationship between TE expression and the regulation of TE-flanking genes is often tested in such pluripotent cells in culture, as they are easier to handle and perturb than early embryos. Although ESCs are not identical to the cells of the inner cell mass (Genet and Torres-Padilla, 2020; Nichols and Smith, 2012), ESCs are characterized by a more open and dynamic chromatin state compared with differentiated cell lines (Meshorer and Misteli, 2006; Gaspar-Maia et al, 2011). In addition, ESCs express a characteristic repertoire of TEs, some of which are also expressed in either the inner cell mass of the blastocyst or morula stage embryo (Grow et al, 2015; He et al, 2019; Kunarso et al, 2010). For example, the ERV element LTR7/HERV-H is considered a pluripotency marker of ‘stemness’ in human ESCs (hESCs) (Carter et al, 2022). Similarly, primate-specific HERVK elements such as LTR5HS, are transcriptionally active in hESCs as well (Grow et al, 2015). Interestingly, perturbation of LTR5HS affects host gene expression, specifically of genes related to stem cell and differentiation, over long genome distances (Fuentes et al, 2018), suggesting once more the involvement of a TE family within a larger gene regulatory network. In fact, there are many reports of LTR expression in ESCs but also of LINEs and SINEs. For example, mouse ESCs express a similar repertoire of LINE-1 elements to those expressed in mouse embryos, and both SINE B1 and B2 elements are also expressed in ESCs (Marks et al, 2012; Fort et al, 2014). Likewise, LINE1 elements are expressed in human ESCs as are Alu SINE elements (Klawitter et al, 2016; Macia et al, 2011; Garcia-Perez et al, 2007; Pal et al, 2023).

Stem cell cultures are heterogeneous and often contain a rare subpopulation of early embryonic-like cells (ECLCs), which display a similar transcriptional profile compared to preimplantation embryos at the timing of genome activation (Rodriguez-Terrones et al, 2018; Macfarlan et al, 2012; Genet and Torres-Padilla, 2020; Taubenschmid-Stowers et al, 2022). In mice, these cells are referred to as 2-cell-like cells (2CLCs), while in humans, they are called 8CLCs, as EGA occurs at the 8-cell stage in human embryos (Braude et al, 1988; Asami et al, 2022). Perhaps unsurprisingly, the LTR mERVL (MT2_mm), which is highly expressed in the 2-cell stage mouse embryo, also becomes highly expressed in 2CLCs (Macfarlan et al, 2012). This is particularly interesting, as in the general population of ESCs, the expression of mERVL is not detectable, and it can therefore be used as a marker of 2CLCs (Rodriguez-Terrones et al, 2018; Macfarlan et al, 2012). Following this finding in mouse ECLCs, human 8CLCs show high expression of LTR elements MLT2A1 and MLT2A2 (Taubenschmid-Stowers et al, 2022). We note that the specific culture conditions of ESCs can dramatically affect the expression levels of TEs and the frequency of ECLCs in the stem cell population (Marks et al, 2012).

The finding that TEs characteristic for EGA are transcriptionally reactivated in ECLCs, further highlights that TEs are a hallmark of the transcriptional program of early embryos. However, whether and if which TEs are drivers of the transcriptional networks in the preimplantation embryo and ECLCs remains to be established.

## Evolutionary pressures on TEs and their host genomes

**Figure 3:**
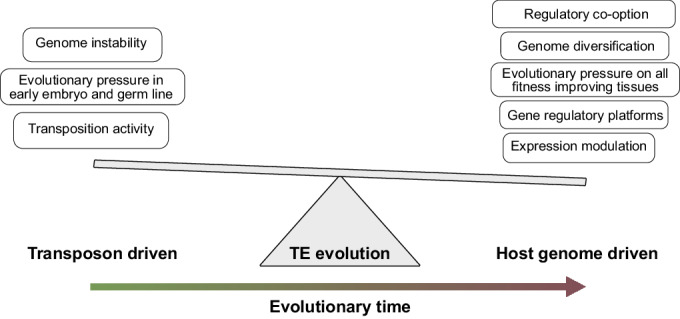
Balancing evolutionary pressures of transposon co-option and genome integrity. Upon insertion in the host genome, TE sequences are exposed to different evolutionary pressures, to both maintain sequence features such as TF binding motifs and TSSs as well as to limit their transposition and transcription potential.

The presence of TEs in eutherian genomes is the result of evolutionary pressure through many millions of generations on both the TE sequences themselves as well as their host genomes (Osmanski et al, 2023). Thus, understanding the product of these evolutionary pressures must be done in the light of a two-faced balance (Fig. 3).

First, there is the TE-driven evolutionary pressure. When the TEs maintain transposition activity, the TE sequences that are most successful in reintegrating in the host genome are able to multiply quickly (Wells and Feschotte, 2020). Although this high transposition rate would be productive for the selfish TE family, it also can result in reduced genome integrity (Belancio et al, 2009; Burns, 2017). Genome instability could in turn lower host survival rate, which would ultimately lead to the disappearance of these highly active TEs from the host population. However, a completely silenced TE would not be able to propagate in its host genome and would therefore only be present at very low numbers in the current host genome. As both retrotransposons and DNA transposons require transcriptional activity for their transposition (Wells and Feschotte, 2020), the evolutionary pressure for the maintenance of transcription and transposition activity go hand in hand.

Second, the maintenance of TE sequences in our genomes is also subject to evolutionary pressure driven by the host genome. Most prominently, the host genome will be more protected against genome instability when the transposition machinery of the TE sequence is impaired as described above. This leads to a higher pressure to lose the sequence integrity of the internal – protein coding – sequences of ERVs, LINEs, and DNA transposons, compared with the terminal sequences such as LTRs, UTRs, and ITRs (Wells and Feschotte, 2020). Interestingly, however, there are several observations in which these TE sequences are used by their host genomes on both a local as well as global level, in particular by co-option of these terminal sequences (Fueyo et al, 2022), as we discuss in more detail below.

Evolutionary pressure can also affect the developmental timing during which TE sequences become active (Sundaram and Wysocka, 2020). In the case of TE-driven evolutionary pressure, in order for the TEs to successfully become inherited by the next generation, transposition has to occur in cells that contribute to the germline; either in the germline itself or early during embryonic development in cells which will give rise to the germline. TEs that propagate after segregation of the germline are not passed on to the next generation. It is therefore not surprising that most transcriptionally active TE sequences are found in the germline and throughout early embryo development (as described above). It has also been hypothesized that regulatory TE co-option could improve the overall fitness of the host, leading to a positive evolutionary pressure in favor of maintaining TE activity beyond the point of germline differentiation. The improved host fitness could in turn lead to a more successful transmission of the genome, including its harbored TE sequences, to the next generation. This could potentially explain why there are TEs expressed and associated with transcriptional regulation of genes in cell types that are not directly inherited by the next generation, such as during later stages of embryo development, the placenta and the immune system (Chuong, 2013; Chuong et al, 2013; Friedli and Trono, 2015; Pontis et al, 2022; Koonin and Krupovic, 2015). Interestingly, these tissues have been associated with rapid evolutionary changes and species diversity, and it has been hypothesized that TEs have contributed to this evolutionary process (Sundaram and Wysocka, 2020).

**Figure 4:**
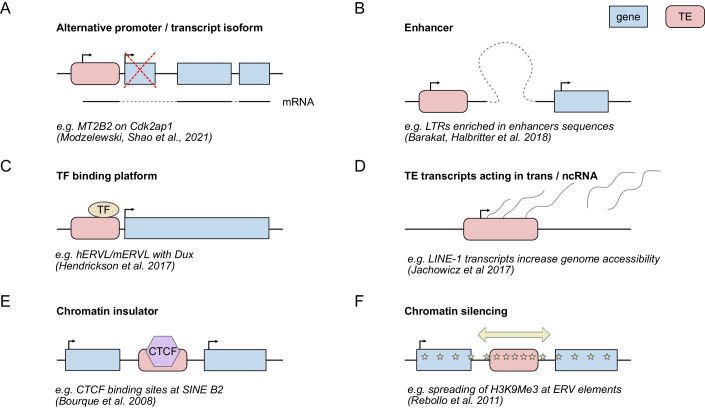
Host genome co-option mechanisms of TE sequences. TE sequences can be co-opted by their host genomes in several different ways, such as using them as alternative promoters for host genes (**A**), as enhancers (**B**), as TF binding platforms (**C**), by TE-derived ncRNA-mediated regulation of genes in trans (**D**), by providing insulator sites (**E**) and by spreading of silencing chromatin marks to neighboring regions (**F**).

The co-option of TE sequences by the host genome is a major source of genome innovation and genome diversification throughout evolution (Fig. 4) (Modzelewski et al, 2022; Fueyo et al, 2022). This is also reflected in the phylogenetic age of different TE families that have been associated with TE co-option. Younger TEs are often associated with species-specific expression and diversification of their regulatory functions and neighboring genes (Sundaram and Wysocka, 2020). One example is the co-option of the young mouse-specific TE, MT2B2, as an alternative promoter that drives transcription of a truncated transcript isoform of the conserved gene *Cdk2ap1* (Modzelewski et al, 2021; discussed in greater detail below). Interestingly, although both gene and transcript isoforms are conserved, different eutherian species seem to co-opt different TEs as alternative promoters for the same gene. Where mouse utilizes the ERVL LTR MT2B2, primates typically co-opt LINE sequences as promoter for the truncated *Cdk2ap1* transcript (Modzelewski et al, 2021). Similarly, the transcription of an isoform of DICER, an essential protein of the RNA interference (RNAi) machinery in mammals, has been shown to be driven by a rodent-specific LTR MT-C in mouse oocyte (Flemr et al, 2013). On the other hand, older TEs are often much more difficult to trace. Over time the TE sequence features dilute and TE families become harder to identify (Storer et al, 2021; Matsushima et al, 2024). However, there are several examples of ancient (evolutionary old) retrotransposons and DNA transposons, which show conserved co-option by host genes and are still active in mammalian genomes (Osmanski et al, 2023; Cosby et al, 2021; Wang and Han, 2020). Notably, a recent study annotated additional ancient TEs using a reconstructed ancestral genome and showed that these ancient TEs contribute to cis-regulatory elements and TE-derived promoters in mammalian genomes, despite being transpositionally dormant (Matsushima et al, 2024). Along these lines, the age of the TE family can also affect when and how individual TE insertions are expressed. Older TEs typically show more variation at an insertion-specific level after being subjected to many generations of evolutionary pressure and accumulation of many site-specific mutations in their sequences (Lanciano and Cristofari, 2020). Younger TEs on the other hand, are typically more similar in sequence within the same family and therefore show more similar expression levels and at similar developmental timing of expression across individual insertions (Lanciano and Cristofari, 2020). In addition to variation that accumulates after insertion on a locus-specific level, TE families can include subgroups with different sequence characteristics, which is independent of the age of the TE family. For example, phylogenetic analysis of individual TE families can further classify them into subfamilies that resulted from waves of transposition activity and are characterized by different transcriptional activity (Franke et al, 2017; Carter et al, 2022). This further highlights that it is essential to understand the evolutionary path of TEs within a host genome when studying the co-option of TEs as regulatory sequences.

## Mechanisms of TE co-option for host genome regulation

As introduced above, both retrotransposon and DNA transposons require transcription in order to jump to new locations in our genome (Wells and Feschotte, 2020). Consequently, TEs have binding sites for the eukaryotic host transcription machinery to bind and initiate transcription. As many repeated occurences of similar TE sequences are distributed throughout the genome, they provide a prime opportunity for the host genome to adapt these sequences as regulatory platforms (Sundaram and Wysocka, 2020). Along these lines, TEs have been implicated in genomic imprinting in the oocyte and early embryo, as well as the placenta, leading to mono-allelic expression (Bogutz et al, 2019; Hanna et al, 2019). There are more examples of host genomes adopting the sequences of TE insertions to regulate transcription on both a local and global genome-wide level (Fueyo et al, 2022), which follow 6 main patterns of genome regulation (Fig. 4).

A classic example of TE co-option on a local level occurs when TE insertions are employed as alternative promoters for host genes, leading to the expression of an alternative transcript isoform during specific times of development or in specific cell lineages (Fueyo et al, 2022; Modzelewski et al, 2021). As introduced above, a recent study by Modzelewski et al, describes an example of this phenomenon, in which an MT2B2 insertion functions as an alternative promoter for the cell cycle gene *Cdk2ap1* specifically during mouse preimplantation development (Modzelewski et al, 2021) (Fig. 4A). This transcript isoform leads to the expression of a truncated CDK2AP1 protein, which the authors show to be essential for preimplantation development in half of the embryos, whereas the other half lacking the truncated protein was born and fertile. During later stages of development after implantation, *Cdk2ap1* switches back to its canonical promoter and a full-length CDK2AP1 isoform is produced (Modzelewski et al, 2021). Similarly, specific insertions of the LTR sequences of MLT2A1 and MLT2A2, which become globally activated in the preimplantation embryo in primates, serve as promoters for a set of protein-coding genes in the pineal gland (Hashimoto et al, 2021). Considering that many TE sequences are located close to, or even within genes in different species (see example in Fig. 1B–D), it is likely that there are more examples for the adoption of TE promoters by genes and transcript isoform switching mediated by TEs yet to be discovered.

A second mechanism by which TEs and their promoters are co-opted is by functioning as enhancers for host genes in cis (Fig. 4B). For example, it has been found that LTR sequences in particular are enriched in enhancers in human ESCs (Barakat et al, 2018). Similarly, many TE sequences still harbor transcription factor (TF) binding motifs, which can result in a local enrichment of TFs at both the TE as well as potentially neighboring genes (Fig. 4C) (reviewed in (Hermant and Torres-Padilla, 2021)). This can positively affect the transcription of these genes (Hendrickson et al, 2017; De Iaco et al, 2017; Whiddon et al, 2017; Gassler et al, 2022; Peaston et al, 2004). A recent study by Pal et al, benchmarked the use of TEs marked by H4K16 acetylation as enhancers in human ESCs, identifying ERV elements as well as LINE1s with cis-regulatory roles (Pal et al, 2023). Both these mechanisms of co-option are particularly intriguing as one can imagine that the simultaneous activation of many repeated insertions of a TE family can regulate networks of genes in this way (Friedli and Trono, 2015; Pontis et al, 2022; Kunarso et al, 2010).

Besides the effect of TEs on neighboring genes in cis, TEs and their transcript products can also affect genes in trans, potentially as non-coding RNA (ncRNA) (Fig. 4D). One example of this is the role of LINE-1 transcripts in regulating global chromatin accessibility in the preimplantation mouse embryo (Jachowicz et al, 2017). The event of transcription and the transcript itself, but not the protein translated from the LINE-1 transcript, affect chromatin accessibility genome-wide. Persistent transcriptional overactivation of the LINE-1 sequences increases global accessibility in embryos at the 8-cell stage, whereas repressing LINE-1 transcription shows a decrease in chromatin accessibility at the 2-cell stage. Noteworthy, the study also shows that both the overactivation and repression affect developmental rates, suggesting that LINE-1s play a role in modulating the appropriate level of chromatin accessibility during early embryonic development in mouse (Jachowicz et al, 2017).

Lastly, TE sequences are not solely associated with positive transcriptional regulation, but have also been suggested to be co-opted as chromatin insulators and repressors of transcription (Fig. 4E,F). In particular, SINE B2 is enriched in CCCTC-binding factor (CTCF) motifs in the mouse genome (Bourque et al, 2008) and ERV sequences are enriched in CTCF binding sites in primate genomes (Schmidt et al, 2012). CTCF is a well-known protein with roles in chromatin architecture that when bound to its motif can block the loop extruder cohesin, resulting in the formation of insulated genomic regions known as topologically associating domains (TADs) (Phillips and Corces, 2009). Interestingly, CTCF motifs within SINE B2 insertions are targeted by SETDB1 in a cell-type-specific manner, thereby modulating appropriate CTCF binding of a given cellular identity (Tam et al, 2024). The finding that certain TEs harbor CTCF sites suggests that the spreading of these TE insertions during evolution also modulated the 3D genome organization.

In addition, TEs can function as hubs of heterochromatin, which can cause spreading of heterochromatic marks to flanking regions (Fig. 4F). As TEs are targeted by the silencing machinery of their host during different development times, they accumulate heterochromatin marks such as H3K9me3 and DNA methylation leading to full transcriptional silencing (Burton and Torres-Padilla, 2014; Almeida et al, 2022; Chitrakar et al, 2022). In some instances, these heterochromatic marks have been shown to spread beyond the TE sequence, thereby affecting the chromatin state of neighboring genes in both human and mouse (Rebollo et al, 2011; Xu et al, 2022; Chitrakar et al, 2022; Yu et al, 2022). Similarly to what we described above, when TE sequences are used directly as promoter and enhancer sequences for host genes these associated genes will become silenced as well at the time when TE expression is repressed by the host during a given developmental time (Rowe et al, 2013; Karimi et al, 2011). Combined, these distinct pathways of TE co-option allow for a complex mode of regulation of the host genome by TEs on both epigenomic and transcriptomic levels, ranging from a small scale at neighboring genes to very large-scale levels of regulation affecting higher-order chromosome organization.

## Concluding remarks

The era of high throughput sequencing not only initiated the study of “junk” DNA in the mouse and human genomes but is also continuously revealing new information about TEs and their remnants harbored in other genomes (see also Box 4). In recent years, we have seen large-scale efforts to assemble the genomes of many mammalian species (Christmas et al, 2023; Upham and Landis, 2023), which allows us to uncover the extent of the conservation and diversity of TEs in their host genomes (Osmanski et al, 2023; Storer et al, 2021). One of the main caveats of TE studies by genomics or transcriptomics is the technical limitations of mapping sequencing data to highly repetitive elements (reviewed in (Lanciano and Cristofari, 2020)). The rapid development of analysis tools enables improved assignment of sequence reads to the correct TE family and even to individual TE insertions (Yang et al, 2019; O’Neill et al, 2020; Jin et al, 2015). Together with the rise of novel genomics techniques that can capture TE transcripts using improved long-read sequencing methods such as nanopore and PacBio (Berrens et al, 2022), this allows for more in-depth study and appreciation of TEs, their evolutionary history and their transcriptional activity (Osmanski et al, 2023; Kirilenko et al, 2023; O’Neill et al, 2020). Lastly, it will be very interesting to investigate the conservation and diversification of the TE families and their co-option in the host genomes of non-model organisms (Osmanski et al, 2023; Storer et al, 2021; Sundaram and Wysocka, 2020). Combined with the advancement of the T2T genomes (Nurk et al, 2022), the next years will see an increasing expansion on our knowledge of TEs, providing the opportunity to reveal molecular and mechanistic function of TEs in genome biology.

Box 4 In need of answers Current literature on TE co-option generally explores the role of a single insertion to a single neighboring gene. However, scientists have hypothesized genome-wide regulatory mechanisms driven by TEs that enable switching ON and OFF large networks of genes. This would be particularly important in early development stages, where thousands of genes are activated in a coordinated fashion (Hermant and Torres-Padilla, 2021; Friedli and Trono, 2015; Rowe and Trono, 2011; Branco and Chuong, 2020). However, whether and if so which specific TFs drive expression of TEs at specific developmental windows and if and how TEs are subsequently driving the expression of entire sets of host genes is still not understood. In addition, most studies focus their efforts on model organisms, particularly mouse and human. TEs however, are clearly not unique to the mouse genome (Rodriguez-Terrones and Torres-Padilla, 2018) and studying the regulatory potential of TEs in different host genomes will uncover the shared as well as species-specific genome innovations that TEs have enabled (Modzelewski et al, 2022). In order to answer these questions, it will be important to understand TEs and their expression patterns on an insertion-specific level (Lanciano and Cristofari, 2020). The advent of long-read sequencing techniques and corresponding computational tools will help enable the accurate mapping of TE transcript to the precise insertion (O’Neill et al, 2020; Jin et al, 2015; Berrens et al, 2022). The advancement of low input and single-cell transcriptomics and genomics techniques allows for the study of the dynamics of transcriptional and epigenetic regulation during the early stages of development, as well as the stochasticity of transcriptional and epigenetic characteristics at TE loci in stem cell populations. Specifically, understanding the interaction of TE sequences within the context of the (epi-)genome will be possible when studying TEs on an insertion-specific level, as highly similar sequences in different genomic neighborhoods may show different expression and epigenetic characteristics. Lastly, the general availability and improved quality of genome assemblies and annotations of non-model mammals opens the possibility to study the roles and rules of transposable elements and genome regulation from an evolutionary perspective (Kirilenko et al, 2023; Osmanski et al, 2023; Christmas et al, 2023; Andrews et al, 2023).

## Glossary

**CTCF**: CCCTC-binding factor
**ECLC**: Early embryonic-like cells
**EGA**: Embryonic genome activation
**ERV**: Endogenous retrovirus
**ESC**: Embryonic stem cell
**ITR**: Inverted terminal region
**LINE**: Long interspersed nuclear element
**LTR**: Long terminal repeat
**ncRNA**: non-coding RNA
**ORF**: Open reading frame
**piRNA**: piwi interacting RNA
**RNAi**: RNA interference
**SINE**: Short interspersed nuclear element
**TAD**: Topological associated domain
**TE**: Transposable element
**TF**: Transcription factor
**TSS**: Transcription start site
**UTR**: Untranslated region
